# Nitric Oxide Enhances Keratinocyte Cell Migration by Regulating Rho GTPase via cGMP-PKG Signalling

**DOI:** 10.1371/journal.pone.0121551

**Published:** 2015-03-23

**Authors:** Rixing Zhan, Shiwei Yang, Weifeng He, Fan Wang, Jianglin Tan, Junyi Zhou, Sisi Yang, Zhihui Yao, Jun Wu, Gaoxing Luo

**Affiliations:** Institute of Burn Research, State Key Laboratory of Trauma, Burn and Combined Injury, Key Laboratory of Disease Proteomics of Chongqing, Southwest Hospital, Third Military Medical University, Chongqing, China; Center for Cancer Research, National Cancer Institute, UNITED STATES

## Abstract

**Objective:**

Nitric oxide (NO) has been shown to improve wound healing, but the mechanism underlying this function is not well defined. Here, we explored the effect of NO on the migration of a human keratinocyte cell line (HaCaT) and its possible mechanism.

**Methods:**

The effects of NO on HaCaT cells in the presence of different concentrations of the NO donor sodium nitroprusside (SNP) were evaluated in a cell migration assay. Subsequently, the cytoskeleton reorganization of cultured HaCaT cells stained with rhodamine-phalloidin was observed with a confocal laser scanning microscope. The mRNA expression and active proteins of CDC42, Rac1 and RhoA in the cultured cells were determined via RT-PCR and pull-down assays, respectively. Furthermore, the roles of various inhibitors or agonists specific to cGMP, PKG and CDC42, Rac1, RhoA in the effects of NO on HaCaT cell migration, F-actin stress fibre formation, and Rho GTPase expression were observed.

**Results:**

It was also found HaCaT cell migration was increased by SNP in a dose-dependent manner, and the other two NO donors either spermine NONOate or SNAP had almost the same effects on HaCat cell migrations. The formation of F-actin stress fibres in SNP-treated HaCaT cells was increased. The mRNA expression and the active proteins of CDC42, Rac1 and RhoA were found to be upregulated after SNP treatment. Similar effects were observed after the cells were treated with a cGMP or PKG agonist. Additionally, the SNP-mediated upregulation of the mRNA expression and the active proteins of CDC42, Rac1 and RhoA were inhibited by the addition of an inhibitor of cGMP or PKG. Moreover, the SNP-mediated promoting effects of migration and cytoskeleton reorganization were inhibited by treatment with inhibitors of cGMP, PKG, CDC42, Rac1 and RhoA respectively.

**Conclusion:**

Our data indicated that the stimulatory effects of NO on cell migration of HaCaT cells are mediated by the cGMP signalling pathway via the upregulation of Rho-GTPase expression, which might promote cytoskeleton reorganization.

## Introduction

Inflammatory mediators produced locally or systemically initiate and regulate cell migration and other processes during wound healing. Many types of inflammatory factors are produced after wounds occur, such as IL-1, IL-3, TNF-α and NO. NO is a small molecule that displays multiple functions in various physiological and pathological processes. Studies have demonstrated that treatment with an NO donor enhances wound healing, but the underlying mechanism is unclear [1,2]. Residual keratinocytes in or around the wound are believed to be one of the crucial cell types responsible for reepithelialization via cell migration and proliferation during wound healing. However, the mechanism initiating the migration of keratinocytes has not yet been elucidated. NO has been found to promote the migration of endothelial cells, bronchial epidermal cells and other cell types [3,4,5,6]. However, the effect and the mechanism of NO action in keratinocyte migration are not well established [7].

Many studies have showed that NO can increase the expression and activation of Rho family members [8,9]. In turn, it has also been found that Small GTP-binding proteins of the Rho family, including RhoA, Rac1, and Cdc42, closely regulate the formation of actin-based structures, cell migration, and proliferation [10,11,12,13]. Rho GTPases are important regulators of cytoskeletal dynamics, and each of the GTPases contributes to cell motility by regulating actin cytoskeletal rearrangements [14]. Continuous reorganization of actin filaments is required for cell mobility and migration, and it has been demonstrated that Rac1 regulates lamellipodia formation, while RhoA regulates actin stress fibre formation and Cdc42 regulates filopodia formation [15]. Therefore, in this study, we investigated the effect of NO on the cultured keratinocyte cell line HaCaT and its possible mechanism through the Rho GTPase signalling pathway. Our primary experiments showed that NO could enhance HaCaT cell migration via the cyclic guanosine monophosphate (cGMP)/protein kinase G (PKG)—Rho GTPase pathway.

## Materials and Methods

### 1. HaCaT cell culture

The HaCaT cell line was obtained from the American Type Culture Collection (ATCC, USA). The cells were cultured in RPMI-1640 medium (Invitrogen, Carlsbad, CA) supplemented with 10% fetal bovine serum (Invitrogen, Carlsbad, CA) at 37°C in a humidified incubator containing 5% CO_2_. The cells were passaged at a ratio of 1:5 using 0.25% trypsin (Sigma-Aldrich, St. Louis, MO) and cultured to 70 to 80% confluence for the following experiments.

### 2. Measurement of cell migration in vitro

An in vitro wound-healing model of cultured HaCaT cells was used according to previous studies [16,17,18] with some modifications. Briefly, HaCaT cells suspended in the above-mentioned medium were seeded in 12-well plates at a density of 2.0×10^5^ cells/2 ml per well and then incubated at 37°C until the cells reached 100% confluence. Mitomycin-C was added at a final concentration of 5 μg per ml, and the cells were incubated for an additional 4 hours to inhibit cell proliferation. A sterile plastic 10-μL pipette tip was used to scratch the confluent cell monolayer evenly in each well to generate a cell-free zone that was approximately 1 mm in width. The medium was aspirated and replaced with fresh RPMI-1640 medium in the absence or presence of different concentrations of sodium nitroprusside (SNP; Sigma-Aldrich, St. Louis, MO). Then, the cells were incubated for 48 hours. The migration of the cells in each well was evaluated under an inverted phase contrast microscope and quantified using ImageJ software (National Institutes of Health, Bethesda, MD, USA) at designated time points, i.e., 0, 6, 12, 24, or 48 hours post-scratch. The migration rates were expressed as (1-the residual area)/(the initial area) × 100%. Each experiment was performed in triplicate. Also, other two NO donors i.e, spermine NONOate (ab144522, abcam) and SNAP (S-Nitroso-N-acetyl-DL-penicillamine, Sigma, Saint Louis, USA) were used to check the possible different effects on HaCat cell migration.

### 3. Observation of the actin cytoskeleton in HaCaT cells

Actin cytoskeleton reorganization was assessed via filamentous actin (F-actin) staining, as reported previously [19]. Briefly, after each treatment, the cells were fixed with 4% paraformaldehyde for 24 hours and permeabilized with 0.2% Triton X-100. Following blocking of nonspecific binding using 0.5% foetal bovine serum, the cells were stained with rhodamine-conjugated phalloidin (100 ng/ml, Biotium, USA) and 4′,6-diamidino-2-phenylindole dihydrochloride (DAPI; 5 μg/mL, Sigma) for 20 minutes. Images of the fluorescently labelled specimens were captured using an LSM 510 laser scanning confocal microscope equipped with immersion lenses (Carl Zeiss Meditec, Jena, Germany). The stress fibres in 100 cells on each slide were randomly analysed under a Zeiss fluorescence microscope. Cells exhibiting stress fibres were analysed with an Olympus fluorescence microscope. LSM 510 software was employed to determine the numbers of cells exhibiting stress fibres according to previous studies [20,21]. The data were expressed as the percentages (mean ± SD) of 100 cells counted on each slide.

### 4. The influence of NO on the active proteins and mRNA expression of CDC42, Rac1 and RhoA in HaCaT cells

#### 4.1 Detection of the active proteins of CDC42, Rac1 and RhoA via pull-down assays

According to the literature, the active proteins of CDC42, Rac1 and RhoA were quantified in pull-down assays [22,23]. After the cells were grown to 70% confluence in RPMI-1640 medium, they were treated with SNP (10 μM) for 10 minutes. The active proteins of CDC42, Rac1 and RhoA were determined via the pull-down method with Activation Assay Biochem Kits (cytoskeleton, Cat. BK034/BK035/BK036, USA) according to the manufacturer's instructions. Briefly, the active proteins of CDC42, Rac1 and RhoA were pulled down by incubating the cell lysates with a fusion protein consisting of the p21-binding domain of Pak1, then separated through SDS-PAGE and detected via Western blot analysis using mouse anti–human CDC42, Rac1 and RhoA antibodies. The total CDC42, Rac1 and RhoA proteins were also measured via Western blotting. The relative quantity of each molecule was calculated from its ratio against the internal reference β-actin.

#### 4.2 Determination of mRNA expression levels via semi-quantitative RT-PCR

At the designated time points, total RNA was extracted from HaCaT cells using TRIzol (Life Technologies, Carlsbad, USA) according to the manufacturer’s instructions and then treated with DNase to decrease genomic DNA contamination. The purity and concentration of the RNA were assayed via electrophoresis and based on the ratio of the optical density at 260 nm to that at 280 nm, respectively.

The Rho family PCR primers and reaction parameters employed in these assays followed Pan and were modified according to our own conditions [24]. The PCR primers and reaction parameters used for Rho family gene amplification are listed in Table 1. The PCR conditions were as follows: initial denaturation at 94°C for 4 minutes, followed by thirty-five cycles of denaturation at 94°C for 30 seconds, annealing at 60°C for 30 seconds and extension at 72°Cfor 60 seconds using a Peltier thermal cycler (Thermo Electron, USA). The appropriate cycles were chosen to assure the termination of PCR amplification during the exponential phase. The gene expression level was presented as the relative yield of the PCR product of the target sequence to that of the internal control, GAPDH. The mean values of three independent experiments were used for analysis.

**Table 1:** Primers and reaction parameters for PCR amplification of human CDC42, Rac1 and RhoA.

Target	Sequence	Annealing temperature (°C)	No. of cycles	Size (bp)
RhoA	5’-CATCCGGAAGAAACTGGT-3’	54	28	168
	5’-TCCCACAAAGCCAACTC-3’			
CDC42	5’-CGATGGTGCTGTTGGT-3’	55	28	312
	5’-CCAACAAGCAAGAAAGGA-3’			
Rac1	5’- AGACGGAGCTGTAGGTAAAA -3’	54	28	236
	5’-ATGCAGGACTCACAAGGGA-3’			

### 5. Roles of cGMP, PKG and Rho-GTPase signalling in the effects of NO on HaCaT cells

Either a cGMP agonist (8-Br-cGMP, 1 μM, Proteinkinase, Germany) or a PKG agonist (8-pCPT-cGMP, 1 μM, Proteinkinase, Germany) was applied to stimulate the HaCaT cells. In some cases, the cells were first pretreated for 2 hours with a cGMP inhibitor (8-Br-cAMP, 1 μM, Proteinkinase, Germany), PKG inhibitor (8-CPT-cAMP, 1 μM, Proteinkinase, Germany), Rho-specific inhibitor, (Rhosin, 30 μM, Calbiochem, USA), CDC42 inhibitor (ZCL278, 30 μM, Calbiochem, USA) or Rac1 inhibitor (Z62954982, 50 μM, Calbiochem, USA). Then, the cells were stimulated with SNP at a final concentration of 10 μM for another 24 hours. Subsequently, cell migration and the actin cytoskeleton were assayed as described in sections 2another respectively. Moreover, the mRNA expression and active proteins of CDC42, Rac1 and RhoA were quantified as described in section 4.1 and section 4.2, respectively.

### 6. Statistical analysis

The data were expressed as the means ± SD of the indicated number of observations. Comparisons between two groups were performed using Student’s t-test. If necessary, one-way analysis of variance (ANOVA) was applied for comparisons among multiple groups. P values < 0.05 were considered statistically significant.

## Results

### 1. Effect of NO on HaCaT cell migration in vitro

**Fig 1:**
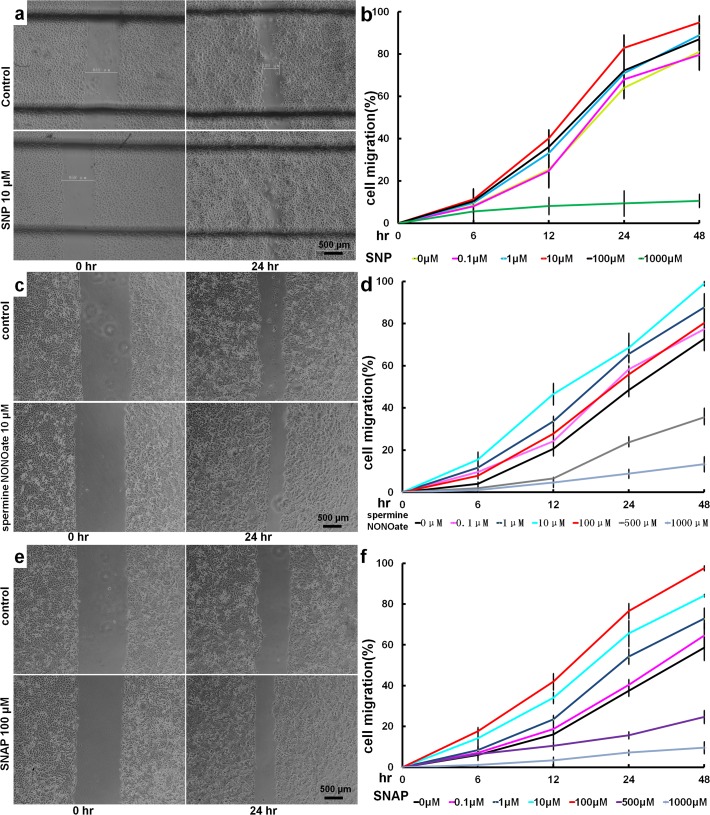
Effect of NO on HaCaT cell migration. An in vitro wound model was developed using cultured sheets of confluent HaCaT cells, as described in the Materials and Methods section. The cells were incubated in the absence or presence of different concentrations of NO donors SNP, spermine NONOate or SNAP. At the designated time points, the cells were imaged under phase contrast microscopy. The closures of the artificial wounds stimulated by SNP (a), spermine NONOate (c) and SNAP (e) were measured. The percentage of the migrated area in the original scratch field was calculated via image analysis and was expressed as the mean ± SD of different wells (b, d, f). Three independent experiments were performed. (b) Quantitative analysis of (a). (d) Quantitative analysis of (c). (f) Quantitative analysis of (e).

As shown in Fig. 1, after treatment with SNP, spermine NONOate or SNAP at different concentrations, cell migrations were increased at the evaluated time points (6, 12, 24 and 48 hours). It was found that the cell migrations were promoted either by SNP, spermine NONOate or SNAP in the range concentrations of 0.1 to 100 μM. Moreover, the concentrations of 10 μM SNP, 10 μM spermine NONOate and 100 μM SNAP were found to be the optimal concentrations for promoting cell migration, respectively (Fig. 1). However, 500 μM of spermine NONOate and SNAP presented an inhibitory effects, and 1,000 μM of spermine SNP, NONOate or SNAP presented a cytotoxicity effect. The results here show that these three NO donors had almost the same effects on the migration of HaCat cells.

### 2. NO-induced reorganization of cytoskeletal actin stress fibres in HaCaT cells

**Fig 2:**
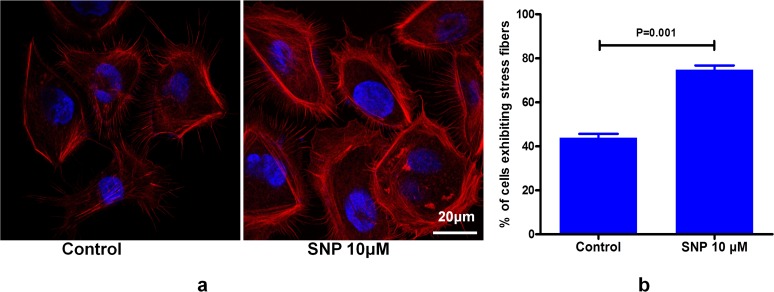
Rhodamine-phalloidin staining of actin stress fibres in HaCaT cells. (a) Representative images of the stimulatory effect of 10 μM SNP on actin stress fibre formation (confocal image magnification = 400x). (b) Statistical analysis of the results. The percentage of cells exhibiting stress fibres (mean ± SD, n = 3 slides) in a total population of 100 cells was calculated for each slide (P< 0.001, compared with the control (Student’s t-test)).

Rhodamine-labelled phalloidin mainly stains polymeric F-actin. As shown in Fig. 2, it was found that SNP could enhance the reorganization of cytoskeletal actin stress fibres in the cultured HaCat cells, with percentage of cells exhibiting stress fibres after SNP treatment being increased by 30.98% compared with the control (n = 300 cells; Fig. 2).

### 3. SNP treatment alters RhoA, Cdc42, and Rac1 expression in HaCaT cells

#### 3.1 SNP impacts the expression of the active and total proteins of CDC42, Rac1 and RhoA in HaCaT cells

**Fig 3:**
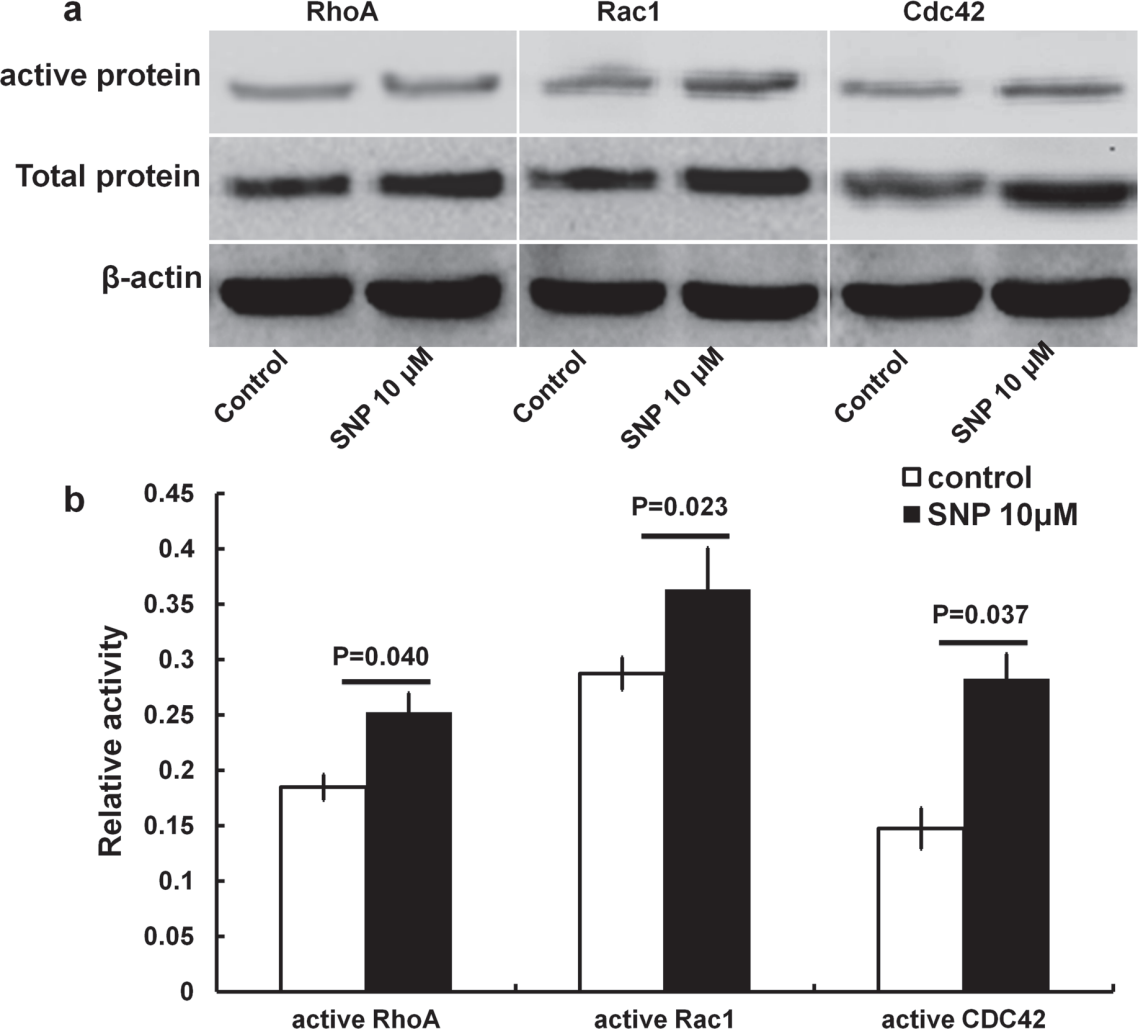
SNP impacts the active and total proteins of CDC42, Rac1 and RhoA in HaCaT cells. The HaCaT cells were treated with or without SNP (10 μM) for 10 minutes. (a) The active and total proteins of RhoA, Rac1 and CDC42 were quantified via the pull-down method. (b) Band intensities of (a) were quantified through densitometry, and the ratio of active RhoA/CDC42/Rac1 to the internal reference β-actin was determined. The presented values are the mean of at least three separate experiments ± SD. (error bars).

Based on pull-down assays, it was found that the expression of the active and total proteins of CDC42, Rac1 and RhoA in the 10 μM SNP treatment group was greater than in the control group (Fig. 3A). The levels of the active proteins of CDC42, Rac1 and RhoA in the 10 μM SNP treatment group were significantly increased, by 13.49%, 7.6% and 6.78%, respectively, compared with the control group (P<0.05) (Fig. 3B).

#### 3.2 Changes in the mRNA expression of CDC42, Rac1 and RhoA in HaCaT cells treated with SNP

**Fig 4:**
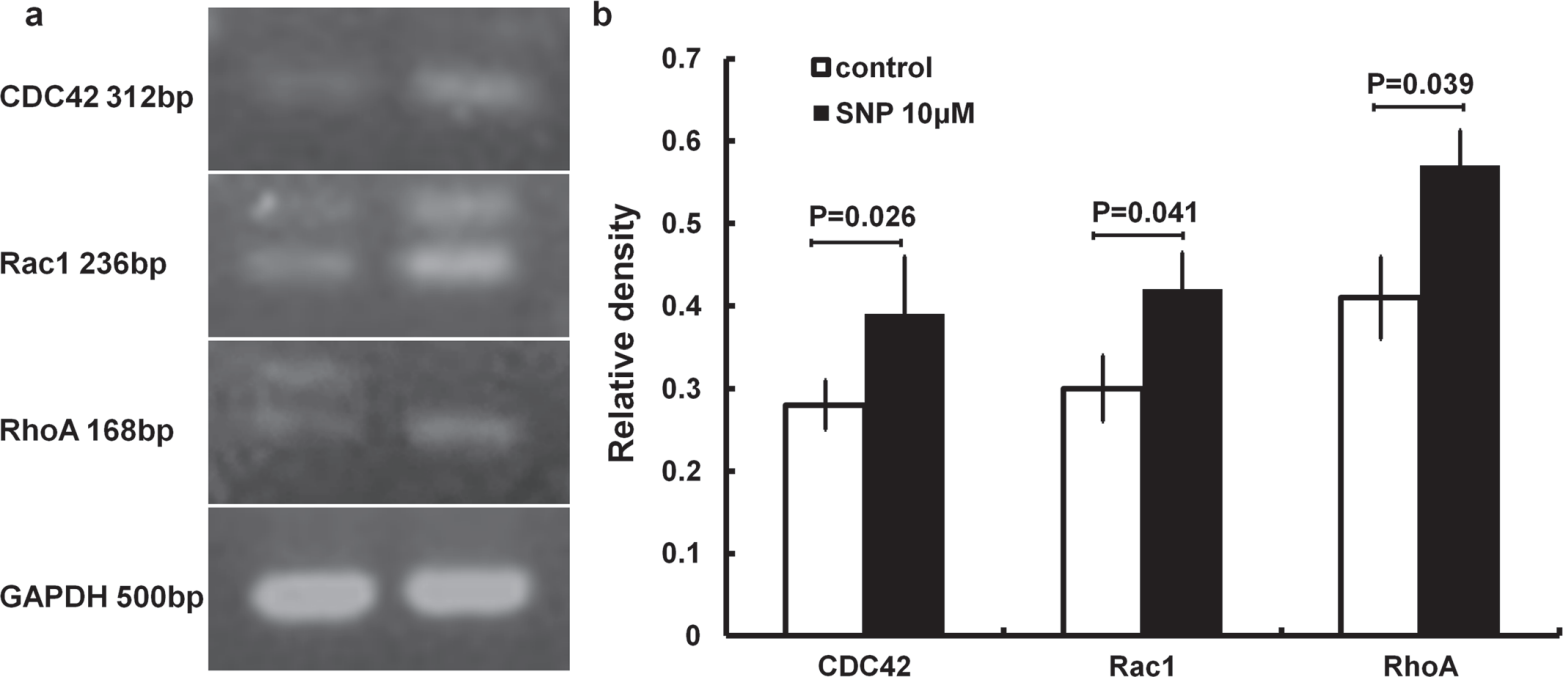
NO promotes the mRNA expression of CDC42, Rac1 and RhoA. (a) HaCaT cells were treated with or without SNP (10 μM) for 24 hours and then subjected to semi-quantitative RT-PCR analysis. (b) Relative densities of CDC42, Rac1 and RhoA.

As shown in Fig. 4, the mRNA expression of CDC42, Rac1 and RhoA in cells treated with 10 μM SNP was significantly augmented compared with control-treated cells (P<0.05) (Fig. 4). The CDC42 mRNA level was increased by 11.26%, Rac1 by 12.21% and Rho A by 15.81% compared with the control group.

### 4. Role of cGMP/PKG signalling in the effects of NO on HaCaT cells

#### 4.1 cGMP/PKG signal transduction is involved in the effect of NO on HaCaT cell migration

**Fig 5:**
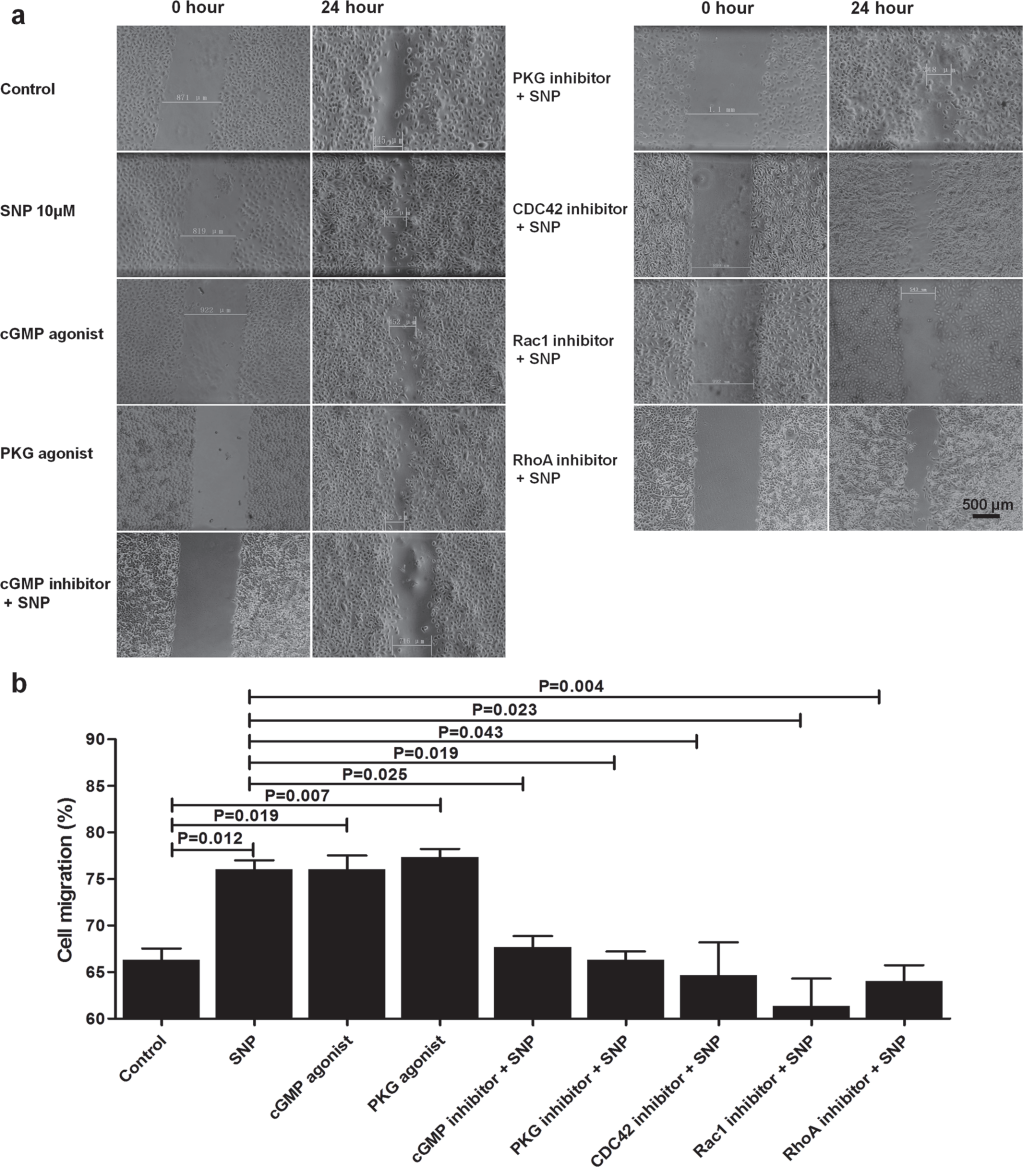
cGMP/PKG signal transduction is involved in the effect of NO on HaCaT cell migration. (a) Treatment with a cGMP agonist (8-Br-cGMP, 1 μM) or PKG agonist (8-pCPT-cGMP, 1 μM) alone. The cells were pretreated with a cGMP inhibitor (8-Br-cAMP, 1 μM), PKG inhibitor (8-CPT-cAMP, 1 μM), Rho-specific inhibitor, (Rhosin, 30 μM), CDC42 inhibitor (ZCL278, 30 μM) or Rac1 inhibitor (Z62954982, 50 μM) for 2 hours, followed by the addition of SNP at a final concentration of 10 μM and incubation for another 24 hours. (b) The results are expressed as the means ± SD of 3 experiments.

The in vitro wound healing assay revealed that, similar to SNP treatment, treatment with either a cGMP or PKG agonist promoted HaCaT cell migration (P<0.05). There was no significant difference between these two groups. Migration was increased by 9.78% and 11.12% in these groups compared with the control group, respectively. However, the effect of SNP on cell migration was significantly decreased by pretreatment with a cGMP, PKG, RhoA, Rac1 or Cdc42 inhibitor (P<0.05) (Fig. 5). Among these treatments, it was found that the inhibition of Rac1 was greatest, decreasing cell migration by 14.7% compared with SNP-treated group.

#### 4.2 NO enhances the reorganization of cytoskeletal F-actin stress fibres in HaCaT cells via cGMP/PKG signal transduction

**Fig 6:**
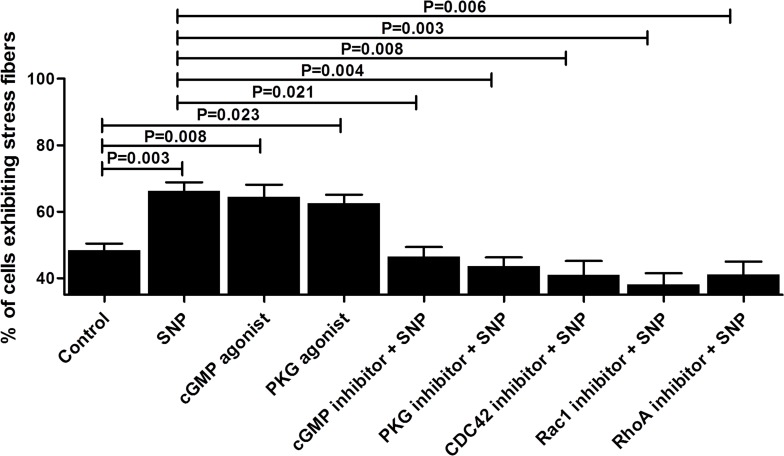
NO enhances F-actin reorganization in HaCaT cells via cGMP/PKG signal transduction. Treatment with a cGMP agonist (8-Br-cGMP, 1 μM) or PKG agonist (8-pCPT-cGMP, 1 μM) alone was able to enhance F-actin reorganization in HaCaT cells. Additionally, the cells were pretreated with a cGMP inhibitor (8-Br-cAMP, 1 μM), PKG inhibitor (8-CPT-cAMP, 1 μM), Rho-specific inhibitor (Rhosin, 30 μM), CDC42 inhibitor (ZCL278, 30 μM) or Rac1 inhibitor (Z62954982, 50 μM) for 2 hours, followed by the addition of SNP at a final concentration of 10 μM and incubation for another 24 hours. The percentage of cells exhibiting stress fibres (mean ± SD, n = 3 slides) in a total population of 100 cells was calculated for each slide.

As shown in Fig. 6, similar to SNP application, treatment with the cGMP or PKG agonist significantly enhanced F-actin reorganization in the cells. Moreover, the SNP-mediated increase in F-actin reorganization was reversed by pretreatment with cGMP, PKG, RhoA, Rac1 and Cdc42 inhibitors to different extents (Fig. 6).

#### 4.3 NO regulates the mRNA expression of CDC42, Rac1 and RhoA in HaCaT cells via cGMP/PKG signalling

**Fig 7:**
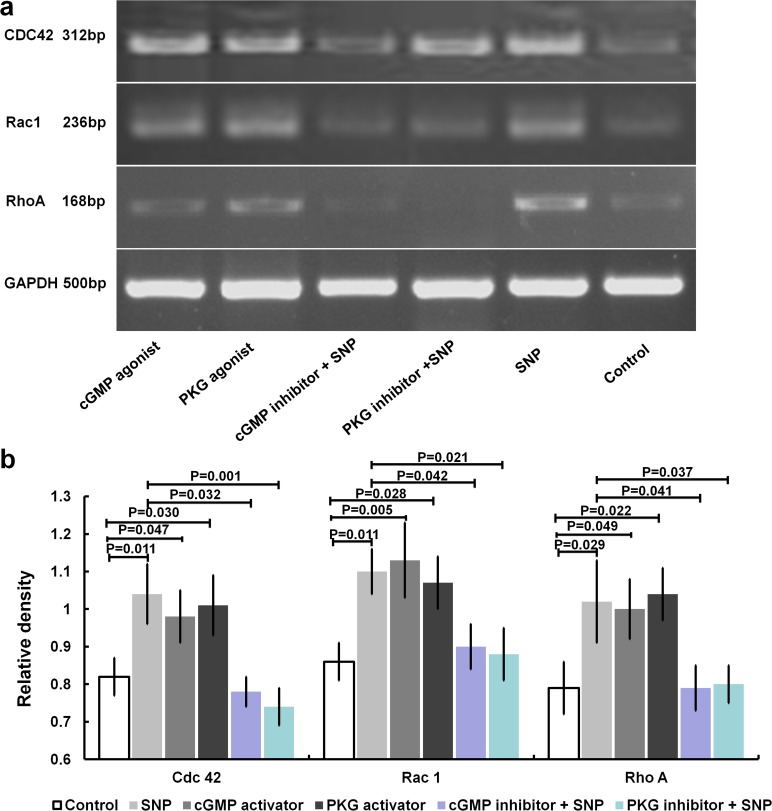
NO upregulates Rho GTPase mRNA expression via cGMP/PKG-mediated signalling. Treatment with 10 μM SNP, a cGMP agonist (8-Br-cGMP, 1 μM) or a PKG agonist (8-pCPT-cGMP, 1 μM) alone was used to stimulate HaCaT cells, or the cells were pretreated with a cGMP inhibitor (8-Br-cAMP, 1 μM) or PKG inhibitor (8-CPT-cAMP, 1 μM) for 2 hours, followed by the addition of SNP at a final concentration of 10 μM and incubation for another 24 hours. (a) The mRNA expression of CDC42, Rac1 and RhoA was quantified via semi-quantitative RT-PCR. (b) The relative levels of CDC42, Rac1 and RhoA mRNA expression were determined by comparison with the internal reference GAPDH.

RT-PCR analysis revealed that, similar to SNP-treated cells, the cells treated with a cGMP or PKG agonist displayed significantly increased mRNA expression of CDC42, Rac1 and RhoA compared with the control cells. Moreover, these effects of SNP were significantly inhibited by pretreatment with a cGMP or PKG inhibitor (Fig. 7). Compared with the SNP-treated group, the expression levels of CDC42, Rac1 and RhoA were decreased by 28.8%, 20% and 17.8%, respectively, in the cGMP inhibitor-treated group and by 31.7%, 18.4% and 19.2%, in the PKG inhibitor-treated group (Fig. 7).

#### 4.4 NO alters the active proteins of CDC42, Rac1 and RhoA in HaCaT cells via cGMP/PKG signalling

**Fig 8:**
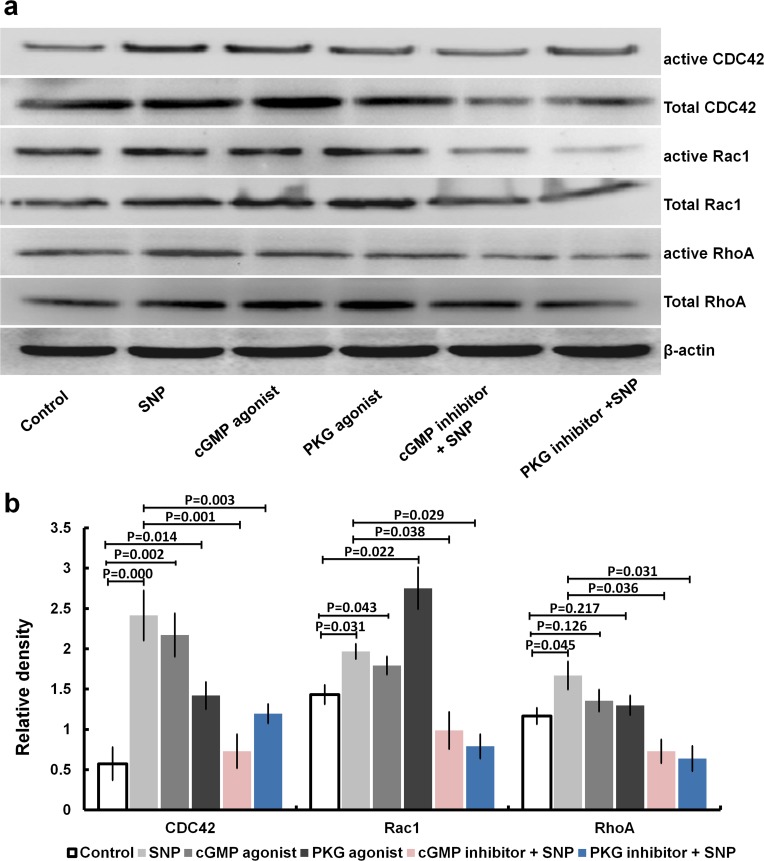
NO alters the activity of Rho GTPases in HaCaT cells via cGMP/PKG signalling. HaCaT cells were treated with SNP (10 μM), a cGMP agonist (8-Br-cGMP, 1 μM) or a PKG agonist (8-pCPT-cGMP, 1 μM) alone for 10 minutes, or the cells were pretreated with a cGMP inhibitor or PKG inhibitor for 10 minutes, then stimulated with 10 μM SNP for 10 minutes. Finally, the total or active proteins of CDC42, Rac1 and RhoA were quantified in pull-down assays (a). The relative ratios of the active CDC42, Rac1 and RhoA proteins against the internal reference β-actin are shown in panels (b).

As shown in Fig. 8, it was found that NO could alter the active proteins of CDC42, Rac1 and RhoA, as determined in pull-down assays. The effects of SNP were significantly inhibited by pretreated with a cGMP or PKG inhibitor versus treatment with SNP (Fig. 8A). Treatment with a cGMP or PKG agonist significantly increased the activity of Cdc42 and Rac1 (Fig. 8B and 8C) and slightly increased the activity of RhoA (Fig. 8D). In addition, the tendency of the changes in the total protein levels of CDC42, Rac1 and RhoA was similar to that of active CDC42, Rac1 and RhoA, respectively, to some extent.

## Discussion

Keratinocyte proliferation and migration are essential for re-epithelization, which is one of the key processes during wound healing, and it has been reported that many factors could affect these processes [25,26]. In this study, it was found that NO can enhance the migration of the cultured keratinocyte cell line HaCaT via a cGMP/PKG—Rho GTPase-mediated pathway.

In our experiments, SNP from Sigma was used as an NO donor, as in many other previous investigations [27,28]. It was observed that in an in vitro wound model, SNP could significantly increase HaCaT cell migration in a dose-dependent manner over a certain range of concentrations. The optimal concentration of SNP for stimulating cell migration was 10 μM. After treatment with 10 μM SNP for 24 hours, the cell migration ratio was increased to 83%, compared with 63% in the control group (p<0.01) (Fig. 1). However, excessive SNP concentrations, particularly 1,000 μM, exerted a toxic effect, which caused the cells to detach, float and even die. In our study, it was found as shown in Fig. 1, the effect trends of the other two NO donors, spermine NONOate and SNAP, on the migrations of HaCat cells are as same as that of SNP (Fig. 1). Other authors have reported that exogenous NO exerts a biphasic effect on cell proliferation [29,30,31]. Frank [29] found that NO exerts a biphasic effect on HaCaT proliferation, with low doses (100 μM) of various NO donors (S-nitrosoglutathione or DETA-NO) mediating a proliferative signal in these cells, whereas high doses (500 μM) induce cytostasis, similar to what was observed in our experiment. A study by Kumar’s [31] revealed the same phenomenon in HL-60 cells. In our study, treatment with low doses of SNP (1–100 μM) resulted in stimulation of cell migration in a dose-dependent manner.

The guanylate cyclase/cGMP/PKG pathway is considered to be the key mechanism by which NO influences cellular functions [32]. NO can directly activate soluble guanylate cyclase, which results in an increased cGMP concentration. Subsequently, cGMP binds to PKG and other target molecules to perform multiple biofunctions [32,33]. In this study, it was found that the migration of cultured HaCaT cells was enhanced by treatment with either a cGMP agonist (8-Br-cGMP) or a PKG agonist (8-pCPT-cGMP). On the other hand, pretreatment with either the cGMP antagonist 8-Br-cAMP or the PKG antagonist 8-CPT-cAMP could abolish the effect of NO on cell migration (Fig. 5). These results indicate that NO influences HaCaT cell migration by activating cGMP and PKG.

Cytoskeletal reorganisation is considered to be a primary mechanism underlying cell migration [34]. In our experiments, it was found that SNP increased the density and quantity of actin stress fibres in cultured HaCaT cells. It has been well demonstrated that small G proteins, especially small Rho family GTPases, participate in the processes of cytoskeletal reorganisation and cell migration. RhoA, Rac1 and Cdc42 are the primary small GTPases that have been found to function in cytoskeletal reorganization [35,36,37]. In this study, it was observed that F-actin in cultured HaCaT cells undergoes reorganisation following treatment with SNP (Fig. 2), whereas treatment with either a RhoA, Rac1 or Cdc42 inhibitor can decrease the effect of SNP (Fig. 6). Babykutty reported that the activity and expression of Rac1 are promoted by NO in a time-dependent manner in colon cancer cells [38]. It was also found that NO could regulate the cytoskeletal architecture, leading to reversible changes in vascular permeability through a Rho GTPase-dependent pathway [9]. A study by Zhou showed that NO drives macrophage migration by modulating the actin cytoskeleton through the small GTPases Cdc42 and Rac1 [39]. Recently, Kiwanuka reported that connective tissue growth factor (CCN2) is able to increase keractinocyte migration through increasing CDC42 activity and decreasing RhoA activity, independent of Rac1 [40]. These data suggested that NO, CCN2 and some other mediators impact cell migration via a different mode of altering Rho GTPase.

Like other members of the Rho small GTPase family, Rho, Rac, and Cdc42are small monomeric G proteins. They cycle between an inactive GDP-bound form and an active GTP-bound form, through which they regulate the actin cytoskeleton, cell migration, proliferation and other cellular processes [41,42,43]. It was demonstrated that Rho can regulate actin polymerization, resulting in the formation of stress fibres and the assembly of focal adhesion complexes. Rac and Cdc42 can induce the formation of filopodia and lamellipodia, respectively, which contribute to the cytoskeletal rearrangements required for cell migration [44].

In the present study, it was found that NO can upregulate RhoA, Rac1 and Cdc42 expression, not only at the mRNA level, but also at the active and total protein levels in HaCaT cells (Figs. 3 and 4). The same effects were observed under treatment with a cGMP or PKG agonist (Figs. 7 and 8). Alternatively, treatment with an inhibitor of cGMP or PKG blocked the stimulatory effect of NO on RhoA, Rac1 and Cdc42 (Figs. 7 and 8). Furthermore, we found that an inhibitor of RhoA, Rac1 or Cdc42 could reduce the enhancement of cell migration by NO (Fig. 6). All of these data indicated that NO facilitates HaCat cell migration by upregulating Rho GTPase via the cGMP and PKG pathways.

In conclusion, the results of our primary experiment suggested that the stimulatory effects of NO on the cell migration of HaCaT cells may be mediated by the cGMP/PKG/Rho GTPase signalling pathway.
